# Cross-Cultural adaption, validity and reliability of a Hindi version of the Corah’s Dental Anxiety Scale

**DOI:** 10.15171/hpp.2018.15

**Published:** 2018-04-18

**Authors:** Meena Jain, Shourya Tandon, Ankur Sharma, Vishal Jain, Nisha Rani Yadav

**Affiliations:** ^1^Manav Rachna Dental College, Faridabad, Haryana, India; ^2^Faculty of Dental Sciences, SGT University, Gurugram, Haryana, India; ^3^Institute of Dental Studies and Technologies, Modinagar, UP, India

**Keywords:** Dental anxiety, Psychometric, Questionnaires, Reliability, Validity

## Abstract

**Background:** An appropriate scale to assess the dental anxiety of Hindi speaking population is lacking. This study, therefore, aims to evaluate the psychometric properties of Hindi version of one of the oldest dental anxiety scale, Corah’s Dental Anxiety Scale (CDAS) in Hindi speaking Indian adults.

**Methods:** A total of 348 subjects from the outpatient department of a dental hospital in India participated in this cross-sectional study. The scale was cross-culturally adapted by forward and backward translation, committee review and pretesting method. The construct validity of the translated scale was explored with exploratory factor analysis. The correlation of the Hindi version of CDAS with visual analogue scale (VAS) was used to measure the convergent validity. Reliability was assessed through calculations of Cronbach’s alpha and intra class correlation 48 forms were completed for test-retest.

**Results:** Prevalence of dental anxiety in the sample within the age range of 18-80 years was 85.63% [95% CI: 0.815-0.891]. The response rate was 100 %. Kaiser-Meyer-Olkin (KMO) test value was 0.776. After factor analysis, a single factor (dental anxiety) was obtained with 4 items.The single factor model explained 61% variance. Pearson correlation coefficient between CDASand VAS was 0.494. Test-retest showed the Cronbach’s alpha value of 0.814. The test-retest intraclass correlation coefficient of the total CDAS score was 0.881 [95% CI: 0.318-0.554].

**Conclusion:** Hindi version of CDAS is a valid and reliable scale to assess dental anxiety in Hindi speaking population. Convergent validity is well recognized but discriminant validity is limited and requires further study.

## Introduction

Dental anxiety is defined as “a patient’s response to stress that is specific to dental situations”.^1^ It is one of the highly prevalent^2-4^ and lesser studied problems^5^associated with dental treatment. Dental anxiety is known to be associated with various dental and general healthcare problems, such as poor quality of life,^6^ abstinence from, or avoidance of dental treatment,^7-9^ as well as lack of sleep.^10^

Generally, dental treatment is related to psychological and physical discomfort. A high percentage of anxious patients are so fearful that they avoid the dental treatment, cancel or delay the appointment, and become less cooperative during dental treatments. Patients may also have a low pain threshold attributable to dental anxiety.^11^ In addition to this avoidance behavior, dental anxiety also has a wide-ranging and dynamic impact on various aspects of a patient’s life.^12,13^ Therefore, an estimation of patient’s anxiety is an important step towards a safe and quality dental care.

Dental anxiety is usually measured using a wide range of available psychometric self-assessment scales such as Corah’s Dental Anxiety Scale (CDAS),^14^ Modified Dental Anxiety Scale (MDAS),^15^ State-Trait Anxiety Scale (STAI),^16^ General Geer Fear Scale,^17^ Getz Dental Belief Survey,^18^ Chotta Bheem-Chutki Scale^19^ and Dental Fear Survey (DFS).^20^ These scales range from 1 item to 20 items or even more. Different scales are based on different theoretical models and they measure dental anxiety from different perspectives.^21^

The CDAS is a 4-item, self-reported measure that asks the participants to rate the anticipated level of anxiety, a day before the visit to the dentist, while sitting in the dentist’s waiting area, when the dentist is preparing the drill, and before scaling of teeth.^14^ In the MDAS, the fifth question concerning local anesthesia injection for dental treatment has been added to CDAS.^15^ However, anxiety over anesthesia may not be directly associated with dental treatment anxiety but could be associated with the use of needles in general. Moreover, not all dental treatments require anesthesia. Therefore, MDAS can overestimate dental anxiety.^22^ Also, the CDAS is probably the oldest and most widely used scale.^22^ The psychometric properties of this scale have been determined in English,^15^ Chinese,^23^ Portugese^24^ and Italian language.^25^ CDAS was also found valid and reliable in a sample of Brazilian adults.^22^

However, translation and validation of this scale have not yet been done for Hindi. According to the Official Languages Act, 1963, Hindi has been adopted as the official language of the Union of India. It is also the largest spoken and understood language by citizens of India, especially of North India. Apart from India, the language is also spoken in many other countries like Nepal, Guyana, Trinidad and Tobago, Suriname, Fiji, and Mauritius. This study was, therefore, conducted to develop and evaluate the validity and reliability of the Hindi version of CDAS when applied to a sample of Hindi speaking adults in India.

## Materials and Methods

The psychometric properties of Hindi version of CDAS were studied through a cross-sectional study, approved by the Institutional Ethics Committee of the institution. The study was conducted from May to June 2017.

The instrument (CDAS) was translated and cross-culturally adapted^26^ in Hindi at an institute of higher dental education in the National Capital Region of India. A “forward and backward blind translation” process was used. Two bilingual professionals who were fluent both in English and Hindi did the forward translation of scale from English to Hindi. These 2 Hindi versions were then again translated into English by 2 other translators, who were not aware of the original English CDAS questionnaire. These back-translated versions were committee reviewed by the authors along with four translators. They reviewed the translations for a comprehensive and semantic equivalence till the Hindi version was considered appropriate by all of them.

This translated version was pretested on the Hindi-speaking population. Twenty subjects from the outpatient department of the dental hospital were selected to fill in the questionnaire. The subjects were then interviewed to find out whether, they understood the translated questions. They were asked to mention if any question or word was difficult to understand. All corrections were discussed among the authors, and appropriate changes were made in the translated Hindi version. The final version was designated as CDAS-H.

A study sample was drawn from the population of patients visiting the outpatient department of the institute. The sample consisted of the patients who visited the dentist earlier, as well as the first time visitors. It included the individuals who consented to participate in the study and who were 18 years or above. Individuals who had either learning, audio-visual, psychiatric or intellectual disability or disorder, as well as those who were unable to read or understand Hindi language were excluded from the study. Validation was done among 348 participants who were selected from the patients attending the Outpatient department (OPD) on the days of study.

The study instrument was the final Hindi version of the 4 item CDAS. The scale measures the perceived dental anxiety of the patients. The respondents filled the self-report questionnaire according to the 5-point Likert scale that ranged from ‘not anxious’ to ‘extremely anxious’. The response was scored from 1 to 5. The scores for all the responses were added to obtain the level of dental anxiety for the participant. The total scores of CDAS for an individual varied from 1 to 20. Basic demographic data like age, gender and education were obtained along with the study instrument.^2,3,4,23,25,27^ Additionally, information such as self-perceived oral health status, previous visit to the dentist and their experience of the visit were also obtained. Apart from the CDAS, a visual analogue scale^27^ (VAS) was also recorded to measure the convergent validity. VAS was taken because no other standard scale to measure dental anxiety has been validated in the Hindi language. VAS has been used in studies for measuring dental anxiety^28^ and was found to be valid.^29^ This was obtained on a scale from 0 to 100 calibrated over a 100 mm line where zero refers to “not at all anxious” and 100 refers to “extreme dental anxiety”. The participant was told to mark a point for the anxiety he felt towards dental treatment. VAS score was assessed by calculating the distance in mm from the left end of the line to the spot where the participant had marked.

Data were analyzed through IBM SPSS^TM^ Statistics for Windows, version 20.0 (IBM Inc., Armonk, NY, USA). Reliability was investigated by calculating Cronbach’s alpha and intra class correlation (95% CI). Sampling adequacy was measured using Kaiser-Meyer-Olkin (KMO) test. Principal Component analysis with Varimax rotation was performed to examine the construct validity and to compare the factorial structure of the Hindi version of CDAS with the original version. Study variables were correlated using Pearson correlation to establish convergent validity. The differences in groups were calculated using independent *t* test (two-tailed) and one-way analysis of variance (ANOVA). Skewness and kurtosis value less than 3 and 7 respectively are considered non-significant deviations from normality and acceptable sensitivity.^30^ All the statistics were considered significant at a *P* value of equal to or less than 0.05.

## Results

### Descriptive statistics

The response rate was 100% as the forms were duly filled. Forty-eight forms that were completely filled for test-retest also had 100% response rate. Male participants in the study were 53%. Table 1 presents the categorization of participants according to their total CDAS score. The prevalence of dental anxiety was 85.63% (95% CI: 0.815-0.891). Around 8% of participants experienced extreme anxiety. The mean CDAS score was 8.73 (SD: 3.55), and the mean VAS score was 52.49 ± (SD: 24.70). None of the participants had CDAS total mean score of 0 or 20.

In the present study, the values of skewness and kurtosis for age, CDAS score, and VAS score were found to be within normal limits. Hence, these values reflect no significant deviation from normality. The age of participants ranged from 18 to 80 years with the mean of 29.98 ± (SD 11.90). Table 2 shows the descriptive statistics of variables evaluated in this study.

### Validity measures 

A positive correlation was observed among four items of CDAS questionnaire in correlation matrix. Bartlett’s test of sphericity was found statistically significant (χ^2^ = 378.747, *P* < 0.0001). The KMO test value was found to be 0.776 which is acceptable to perform factor analysis. Eigen value for the single factor (dental anxiety) was 2.441 which demonstrated 61% of the variance (Table 3).

### Discriminant Validity

*Age*

CDAS total mean score of participants of age ≤30 years, 31-50, and ≥51 was 8.70, 8.87, and 8.54 respectively. One-way ANOVA showed that this difference was not significant statistically (*F* value: 0.113) (Table 2). Also, very weak correlation was found between age and CDAS total mean score, which was statistically not significant (*r*= -0.002, *P* > 0.05).

*Gender*

Mean score of CDAS for males was 8.85 whereas it was 8.60 for females but the difference was not statistically significant (Table 2). Kendall’s Tau Correlation Analysis showed no significant relation of gender with CDAS total score (*r* = -0.027, *P* > 0.05).

*Education*

The CDAS total mean scores according to the level of education are presented in Table 2. Participants with primary level of education had highest mean CDAS score. But one-way ANOVA showed that the difference in scores was not statistically significant (*F* value: 1.947).

*Dental attendance*

The mean CDAS score of patients who had earlier visited a dentist was 8.43 whereas for those who had not visited any dentist was 9.25 and the difference was statistically significant (*t* test: 2.081). From the 220 participants who had visited a dentist earlier, 183 had a good experience with the mean CDAS score of 7.87 which is less than CDAS score 11.22 of those who had a bad experience during their dental visits. The difference was statistically significant (*t* test: 5.691) (Table 2).

*Oral Health*

CDAS total mean score was highest (10.67) for the participants who had a poor self-perceived oral health status. These scores decreased as the self-perceived oral health status improved. One-way ANOVA showed that this difference was highly significant statistically (*F:* value 5.653, *P* < 0.001) (Table 2).

### Convergent validity

Pearson correlation coefficient was calculated to assess the convergent validity of the Hindi version of CDAS. Pearson correlation coefficient of the individual items CDAS 1, CDAS 2, CDAS 3, and CDAS 4 with VAS score was 0.327, 0.352, 0.407, and 0.466 respectively. CDAS and VAS scores had a highly significant correlation (*r* = 0.494, *P* <0.001), indicative of a fairly positive correlation.

### Reliability measures

The inter-item correlations among 4 items in consecutive visits among 48 participants were to be found positive. Cronbach’s alpha for the test-retest was 0.814. Correlation among 4 CDAS items in first and second visit using Pearson correlation was 0.733, 0.737, 0.570, and 0.493 for CDAS 1, CDAS 2, CDAS 3, and CDAS 4, respectively (*P* <0.001). A positive correlation was observed among 4 items in the sample of 348. The Intra-class correlation coefficient between four items was 0.425 (95% CI: 0.318-0.554, *F* test: 8.397, *P*<0.001). Cronbach’s alpha for 348 study subjects was 0.782. Table 4 also details the inter-item statistics which showed that all the four items contributed significantly and the internal consistency of the CDAS scale is acceptable.

Table 5 presents the intra-class correlation coefficient values for test-retest of 4 individual items refilled by 48 study subjects after 15 days. The test-retest ICC of the total CDAS score was 0.881 (95% CI: 0.318-0.554) with a *P* value <0.001 indicative of an excellent agreement.

## Discussion

It is vital for a dentist to assess the dental anxiety levels of a patient so as to provide a good dental experience. It is also suggested to assess dental anxiety before preventive programs for better patient participation in oral hygiene maintenance. A valid and reliable scale to evaluate the dental anxiety of Hindi speaking Indian population was lacking. Hence, this hospital-based cross-sectional study was conducted in India to develop an appropriate Hindi version of CDAS and to evaluate the psychometric properties of the translated instrument. The present study results showed that the CDAS-H has good psychometric properties. The convergent validity (Pearson correlation coefficient = 0.494) was good. In this study, factor analysis resulted in loading on one factor which was consistent with observations from studies in Portuguese^24^ and Brazilian^22^ populations.

Cronbach’s alpha value of Hindi CDAS in the present study was 0.78 which is good and acceptable. The value of Cronbach’s alpha for adults from Brazilian population was 0.83,^22^ from Portuguese population was 0.838,^24^ for Italian population was 0.883^25^ and from English population, it ranged from 0.75 to 0.92 in various subgroups.^15^ The items fitted well with each other on the scale because the corrected item-total correlation coefficients of all 4 items were above 0.4.^31^ Also, CDAS-H has excellent test-retest reliability (ICC = 0.881). The 100% response rate for this questionnaire also indicates that a nominal supervision is required for measuring dental anxiety using CDAS-H. Also, the floor and ceiling effect for CDAS-H was not present.

In the present study, 8% of the participants had extreme dental anxiety. This was higher as compared to other studies done in Indian population by Acharya^32^(2.2%), Appukuttan et al^2,3^(3%), Appukuttan et al^33^(2.7%) and Marya et al^34^(4.4%). However, the percentage was lesser than in studies conducted in other countries like USA^4^ (20%), Bulgaria^35^ (11.7%), UK^36^ (11%), Turkey^37^ (23.5%) and Iran^38^ (12.5%). This may be due to difference in cultures and ethnicity of various study populations.

The present study showed no relation between age and mean dental anxiety score which was similar to the study done in Gujrati,^39^ Portuguese^24^ and Iranian^38^ populations. However, the studies in Indian population by Acharya,^32^ Appukuttan et al^2,3,33^ and Marya et al^34^ showed that dental anxiety reduced as the age increased. Further, studies from other countries like China,^23^ USA^4^ and Bulgaria^35^ also showed an inverse relation between age and dental anxiety level. However, Tunc et al^37^ showed a positive relation between age and dental anxiety. Therefore, the association between age and dental anxiety is not clear.

Most of the studies showed that females have higher mean dental anxiety scores as compared to males.^25,32,33,37-39^ It is believed that females acknowledge their anxiety more easily compared to their male counterparts.^27^ However, the present study showed that dental anxiety scores are independent of gender and the result was similar to the studies in Nepali^31^ and in Portuguese^24^ populations. There was no significant effect of education on dental anxiety levels in the present study sample and this result is consistent with the study in Portuguese^24^ population.

In this study, the self-perceived oral health status was inversely related to mean CDAS scores. The results were similar to study by Appukuttan et al.^33^ Participants with good previous dental experience had lesser mean dental anxiety scores in comparison to patients with a bad previous experience at a dental visit and this result was similar to the study conducted by Acharya.^32^

Several studies have shown a positive relationship of dental anxiety with general anxiety and depression.^40,41^ An association has been found between psychological status and dental anxiety.^42^ Patients with dental anxiety can have some underlying psychological distress which needs to be addressed. In patients with extreme dental anxiety, therefore, a dentist should motivate the patient to consult mental care professionals.^41^ CDAS-H is helpful for both dental and mental health professionals to assess dental anxiety during treatment.

Both, a good sample size of 348 subjects and a wide range of age group of participant’s increases the generalizability of results from the present study. The limitation of this study was that it used a self-reported questionnaire; therefore, dental anxiety levels in uneducated population could not be studied. Secondly, being a hospital-based study; it could have underestimated the prevalence of dental anxiety and percentage of extremely anxious subjects. Thirdly, criteria validity could not be established due to the absence of standard scales to measure dental anxiety/fear in Hindi language. Fourthly, a confirmatory factor analysis in a larger sample is warranted. Despite the fact, this study has made novel efforts in cross-cultural reliability and validity of CDAS-H.

## Conclusion

The CDAS-H showed acceptable levels of reliability and validity. Convergent validity was well established as VAS correlated significantly with total CDAS score as well as with each item of CDAS individually. However, discriminant validity requires further studies as factors influencing or determining dental anxiety are still not established in Hindi speaking population. Also, not many studies have measured dental anxiety in Hindi speaking population. Epidemiological studies using CDAS-H are required to assess dental anxiety at the state or national level. CDAS-H can be utilized for research purposes as well as for individual patients in the dental office for those having Hindi as their first language. Further, it is recommended to translate and validate CDAS in various other languages for use in populations with different cultures and languages.

## Ethical approval

Ethical approval to conduct the study was taken from the Institutional Ethics Committee, Manav Rachna Dental College, Faridabad, Haryana, India, with letter reference number MRDC/IEC/2017.280.

## Competing interests

The authors declare that there is no conflict of interest.

## Authors’ contributions

MJ involved in the conception and designing the study, data interpretation, wrote manuscript and acted as corresponding author. ST involved in the conception and designing the study, supervised the development of work, evaluated and edited the manuscript. AS performed the translation of instrument, data analysis and interpretation, evaluated and edited the manuscript. VJ involved in the conception and designing the study, data analysis, helped in writing the manuscript, evaluated and edited the manuscript. NRY performed the translation of instrument, helped in data collection, to evaluate and edit the manuscript.

## Acknowledgments

The authors wish to express their sincere gratitude to all those who motivated and helped them in conducting this study.

**Table 1:** Categorization of patients based on CDAS scores

**CDAS score range**	**No. (%)**	**Mean CDAS score**
0-4 (not anxious)	50 (14.37)	4
5-8 (low anxiety)	131 (37.64)	6.54 ± 1.10
9-12 (moderate anxiety)	109 (31.32)	10.49 ± 1.12
13-14 (high anxiety)	30 (8.62)	13.33 ± 0.48
15-20 (extreme anxiety/phobic)	28 (8.05)	15.68 ± 0.61
Total	348 (100)	8.73 ± 3.55

**Table 2:** The variables with the percentages, mean total score and statistical test

**Variable**	**No. of samples**	**Percent**	**Mean total CDAS score ± (SD)**	***P*** ** value**
Age group				>0.05
≤30	231	66.38	8.70 ±****(3.55)	
31-50	91	26.15	8.87 ±****(3.57)	
≥51	26	7.47	8.54 ±****(3.59)	
Gender				>0.05
Male	185	53.2	8.85 ±****(3.55)	
Female	163	46.8	8.60 ±****(3.56)	
Education				>0.05
Primary level	57	16.4	9.49 ±****(3.59)	
Senior Secondary level	65	18.7	8.03 ±****(3.56)	
Degree/Diploma	200	57.5	8.69 ±****(3.55)	
Post-graduation	26	7.5	9.23 ±****(3.58)	
Oral Health				<0.001
Excellent	57	16.4	7.43 ±****(3.56)	
Good	163	46.8	8.68 ±****(3.55)	
Average	101	29	9.03 ±****(3.57)	
Poor	27	7.8	10.67 ±****(3.79)	
Visited earlier to dentist				<0.05
Yes	220	63.2	8.43 ±****(3.55)	
No	128	36.8	9.25 ±****(3.57)	
Previous dental experience				<0.001
Good	183	83.2	7.87 ±****(3.55)	
Bad	37	16.8	11.22 ±****(3.56)	

**Table 3:** Exploratory factor analysis with rotation

**Component**	**Initial Eigen value**	**Percentage of variance**	**Percentage** **Cumulative**
1	Total= 2.441	61.019	61.019
Items	Matrix of the factorial structure		Total Communalities
Q1	0.755		0.570
Q2	0.796		0.634
Q3	0.789		0.623
Q4	0.783		0.614
Extraction method- Principal Component Analysis with Varimax rotation			

**Table 4:** Item-total statistics

	**Scale mean if item deleted**	**Scale variance if item deleted**	**Corrected item-total correlation**	**Cronbach's alpha if item deleted**
Q1	6.57	7.012	0.562	0.750
Q 2	6.66	7.850	0.615	0.717
Q 3	6.55	7.931	0.598	0.725
Q 4	6.41	7.673	0.593	0.726

**Table 5:** Intra-class correlation coefficient (ICC) values for test-retest reliability of the 5 items and total score of Hindi version of CDAS

**DAS**	**ICC**	**95% CI**		***F*** ** test**	***P*** ** value**
		**Lower**	**Upper**		
CDAS 1	0.839	0.713	0.910	6.206	<0.001
CDAS2	0.842	0.719	0.912	6.337	<0.001
CDAS 3	0.726	0.511	0.846	6.486	<0.001
CDAS 4	0.660	0.393	0.809	2.938	<0.001
Total CDAS	0.881	0.824	0.925	8.397	<0.001
